# Aberrant Expression Profile of Long Noncoding RNA in Human Sinonasal Squamous Cell Carcinoma by Microarray Analysis

**DOI:** 10.1155/2016/1095710

**Published:** 2016-12-01

**Authors:** Ling-zhao Meng, Ju-gao Fang, Jing-wu Sun, Fan Yang, Yong-xiang Wei

**Affiliations:** ^1^Department of Otolaryngology-Head and Neck Surgery, Beijing Anzhen Hospital, Capital Medical University, Beijing 100029, China; ^2^Department of Otolaryngology-Head and Neck Surgery, Beijing Tongren Hospital, Capital Medical University, Beijing 100730, China

## Abstract

*Objectives.* This study aimed to identify aberrantly expressed long noncoding RNAs (lncRNAs) profile of sinonasal squamous cell carcinoma (SSCC) and explore their potential functions.* Methods*. We investigated lncRNA and mRNA expression in SSCC and paired adjacent noncancerous tissues obtained from 6 patients with microarrays. Gene ontology (GO) analysis and pathway analysis were utilized to investigate the gene function. Gene signal-network and lncRNA-mRNA network were depicted. Quantitative real-time polymerase chain reaction (qRT-PCR) was utilized to validate 5 lncRNAs in a second set of paired SSCC and adjacent noncancerous tissues obtained from 22 additional patients.* Results.* We identified significantly differentially expressed lncRNAs (*n* = 3146) and mRNAs (*n* = 2208) in SSCC relative to noncancerous tissues. The GO annotation indicated that there are some core gene products that may be attributed to the progress of SSCC. The pathway analysis identified many pathways associated with cancer. The results of lncRNA-mRNA network and gene signal-network implied some core lncRNAs/mRNAs might play important roles in SSCC pathogenesis. The results of qRT-PCR showed that all of the 5 lncRNAs were differentially expressed and consistent with the microarray results.* Conclusion*. Our study is the first screening and analysis of lncRNAs expression profile in SSCC and may offer new insights into pathogenesis of this disease.

## 1. Introduction

Head and neck squamous cell carcinoma is the sixth most common malignancy worldwide. Sinonasal squamous cell carcinomas (SSCC) are rare tumor, estimated to account for approximately 3–6% of all head and neck squamous cell carcinomas. SSCC originate in the respiratory epithelium of the sinonasal cavities [1]. Approximately 60% of SSCC arise in the maxillary sinus, 20–30% in the nasal cavity, 10–15% in the ethmoid sinuses, and ~1% in the frontal and sphenoid sinuses [2, 3]. Environmental factors, such as wood dust and textile, may play a critical role in the development of SSCC. As in other head and neck squamous cell carcinomas, smoking is a known risk factor [4]. Males who have greater occupational exposure to carcinogens are affected twice as often as females. It is controversial that chronic inflammatory sinus disease could influence development of SSCC. Human papilloma virus (HPV) types 16 and 18 may be implicated in malignant transformation of inverted papillomas [5]. The low incidence of SSCC combined with their nonspecific symptoms often leads to a critical delay in diagnosis. Treatment for SSCC is usually primarily surgical with adjuvant radiotherapy and sometimes with adjuvant chemotherapy for all except small tumors [1]. Surgical management of SSCC is of great challenge due to its anatomical complexity, especially advanced SSCC involving eye, skull base, or infratemporal fossa. In spite of major advances in the therapy of SSCC, including surgery and chemoradiotherapy methods, the 5-year survival rate is still very low (30–50%) [1].

Long noncoding RNAs (lncRNAs) are a subset of noncoding RNAs >200 nucleotides in length and do not encode any protein. Due to the poor evolutionary conservation relative to the protein coding regions of the genome, lncRNAs were once considered as transcriptional noise or junk and have not been well studied historically. Recently, considerable evidence has been accumulating showing that aberrant expression of lncRNAs contributes to the development of human cancers [6–8]. lncRNAs contribute to tumor development through numerous different cellular processes, ranging from transcriptional and posttranscriptional regulation of relevant genes to the control of cell cycle distribution, cell differentiation, and epigenetic modifications. lncRNAs may be involved in cell proliferation, tumor invasion, metastasis, or apoptosis process. lncRNAs are pervasively transcribed and have a critical role in genome regulation [6, 7, 9]. However, to our knowledge, little is known about lncRNAs expression profile in SSCC, and the potential pathways regulating SSCC invasiveness remain poorly understood.

This pilot study aimed to identify aberrantly expressed lncRNAs profile of SSCC and explore their potential functions. This study will help us to understand the tumorigenesis and development of SSCC and provide some new biomarkers that may be critical to the developmental cascade.

## 2. Materials and Methods

### 2.1. Patients and Tissue Samples

A total of 28 pairs of primary SSCC tissues and their paired adjacent noncancerous sinonasal tissues were surgically obtained from adult patients undergoing treatment at Anzhen Hospital and Tongren Hospital (two tertiary academic centers in Beijing, China) between January 2013 and August 2014. During surgery, fresh tumor tissue and paired noncancerous tissue isolated from at least 2 cm away from the tumor border (sometimes contralateral normal sinonasal mucosa) were collected in the operating room and processed immediately in liquid nitrogen within 15 minutes and then stored in RNA Fixer Reagent (Bioteke, Beijing, China) at −80°C prior to total RNA extraction. 6 pairs of tissues underwent microarray analysis (Table 1) and the remaining 22 tissues were used in validation studies by quantitative real-time polymerase chain reaction (qRT-PCR). Tobacco smoke was the most common exposure factor, about 66.67% (4/6) in the microarray analysis series and 63.64% (14/22) in the PCR validation series. Wood dust was the second common exposure factor, about 33.33% (2/6) in the microarray analysis series and 18.18% (4/22) in the PCR validation series. Other exposure factors were chronic sinusitis (3/22) and leather dust (1/22). All cases were reviewed by two or more independent pathologists, and none of the patients had been previously treated with radiotherapy or chemotherapy. All tumor staging was determined according to the tumor-node-metastasis (TNM) staging criteria of American Joint Committee on Cancer (AJCC), 2010.

The Ethics Committee in Clinical Research of Capital Medical University approved this study, and written informed consent was provided by all patients.

### 2.2. Transcript Analysis

RNA extraction was carried out using standard methods (Life Technologies; RNA Easy, Qiagen, Valencia, CA, USA). Total RNA was quantified by the NanoDrop ND-2000 (Thermo Scientific) and RNA integrity was assessed using Agilent Bioanalyzer 2100 (Agilent Technologies).

Microarray profiling was conducted with the Agilent Human lncRNA (4*∗*180 K, Design ID: 062918) in this experiment and data analysis of the 12 samples has been completed in the laboratory of the KPS Biotechnology Company in Beijing, China. The sample labeling, microarray hybridization, and washing were performed based on the manufacturer's standard protocols. Briefly, total RNA was transcribed to double strand cDNA and then synthesized into cRNA and labeled with Cyanine-3-CTP. The labeled cRNAs were hybridized onto the microarray. After washing, the arrays were scanned with the Agilent Scanner G2505C.

Feature Extraction software (version 10.7.1.1, Agilent Technologies) was used to analyze array images to obtain raw expression data, which was processed using GeneSpring. Briefly, raw data was normalized with the quantile algorithm. Probes which had at least 1 out of 2 conditions having 75% flags in “*p*” were selected for further data analysis. Differentially expressed gene transcripts were later identified. We set a standard threshold set for up- and downregulated genes of a fold change ≥2.0 and a *p* value ≤0.05.

Hierarchical clustering was performed to display expression patterns among samples. Briefly, we calculated the distance matrix between the gene expression data. Once this matrix of distances was computed, clustering begins. Agglomerative hierarchical processing consisted of repeated cycles where the two closest remaining items (those with the smallest distance) are joined by a node/branch of a tree, with the length of the branch set to the distance between the joined items. The two joined items were removed from the list of items being processed and replaced by an item that represents the new branch. The distances between this new item and all other remaining items were computed, and the process was repeated until only one item remained.

### 2.3. lncRNA-mRNA Coexpression Networks

*R* function cor. test (a test for association/correlation between paired samples) was utilized to compute Pearson's correlation coefficient to measure the gene coexpression. The lncRNAs/mRNAs (Pearson correlation coefficients ≥0.93) were selected to draw the network with Cytoscape.

According to these data, we built lncRNA-mRNA network using the correlation coefficients to examine interactions between lncRNA and mRNA. The value of “degree” in coexpression network indicated that one mRNA/lncRNA might be correlated with several lncRNAs/mRNAs.

### 2.4. GO Analysis and KEGG Pathway Analysis

GO analysis was applied to analyze the main function of the differential expression genes according to the GO database. Pathway analysis was used to find out the significant pathway of the differential genes according to KEGG. We used Fisher's exact test and *χ* ^2^ tests to select the significant pathway, and the threshold of significance was defined by *p* value and false discovery rate (FDR). The enrichment Re was calculated using standard methods with a *p* value (hypergeometric-*p* value) denoting the significance of the pathway correlated with the conditions, with a threshold of *p* < 0.05, adjusted for multiple comparisons.

### 2.5. Gene Signal-Network

Gene-gene interaction network was constructed based on the data of differentially expressed genes. Java was utilized to build and analyze molecular networks. After parsing the whole KEGG database, selected genes involved in relevant pathways were extracted, and the study pathway network was generated with the help of the pathway topology in the KEGG database.

### 2.6. qRT-PCR Analysis

Total RNA was extracted and purified using standard methods (Life Technologies; RNA Easy, Qiagen, Valencia, CA, USA). M-MLV reverse transcription (Promega) was utilized to synthesize cDNA. 5 lncRNA expressions in sinonasal tissues were measured by qRT-PCR which was performed on the ABI 7500 qPCR system with the primer pairs listed in Table 2. The raw quantifications were normalized to the beta-actin gene values for each sample and fold changes were shown as mean ± SD in three independent experiments, each in triplicate.

### 2.7. Statistical Analysis

All data were expressed as the mean ± SD or proportions where appropriate. Expression levels between SSCC tissues and adjacent nontumor tissues were analyzed by paired-sample *t*-tests. *p* values <0.05 (two-tailed) indicated statistical significance. The Statistical Program for Social Sciences (SPSS) 21.0 software (SPSS, Chicago, IL, United States) was employed to perform all of the statistical analyses.

## 3. Results

### 3.1. Overview of lncRNA Profile

**Figure 1:**
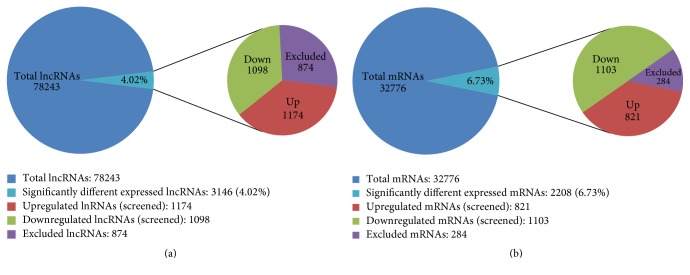
(a) Brief microarray results of lncRNAs. Expression levels of 78,243 lncRNAs were assessed in 6 pairs of SSCC tissues and paired adjacent noncancerous sinonasal tissues using Agilent Human lncRNA 4*∗*180 K microarrays. Compared with paired adjacent noncancerous tissues, 3146 lncRNAs (4.02%) had significant changes in expression levels (fold change >2, *p* < 0.05). A total of 874 lncRNAs were excluded due to low expression levels. A total of 3146 lncRNAs were then identified from the screen, with 1174 upregulated and 1098 downregulated. (b) Brief microarray results of mRNAs. Expression levels of 32,776 mRNAs were assessed in 6 pairs of SSCC tissues and paired adjacent noncancerous sinonasal tissues using Agilent Human lncRNA 4*∗*180 K microarrays. Compared with paired adjacent noncancerous tissues, 2208 mRNAs (6.73%) had significant changes in expression levels (fold change >2, *p* < 0.05). A total of 284 mRNAs were precluded due to low expression levels. A total of 2208 lncRNAs were then identified from the screen, with 821 upregulated and 1103 downregulated.

Out of a collection of 78,243 lncRNAs and 32,776 mRNAs probes, our lncRNA expression profile of 6 malignant sinonasal tissue and corresponding normal tissue samples from patients with SSCC indicated dysregulation of 6.73% (821 upregulated and 1103 downregulated transcripts) of mRNA and 4.02% (1174 upregulated and 1098 downregulated transcripts) of lncRNA transcripts in SSCC tissues (fold change >2, *p* < 0.05) (Figure 1). As expected, the lncRNA and mRNA expression profiles allowed distinguishing malignant and normal tissue samples accurately based on the molecular signature.

Out of the group of RNAs that were upregulated, lncRNA NONHSAT096777 and mRNA HORMAD1 showed the greatest degree of demonstrated upregulation, with 212.076- and 91.757-fold increases, respectively; of those that were downregulated, lncRNA TCONS_l2_00002973 and mRNA ANKRD30A demonstrated the greatest degree of downregulation, with 298.204- and 275.902-fold decreases, respectively (Tables 3 and 4).

**Figure 2:**
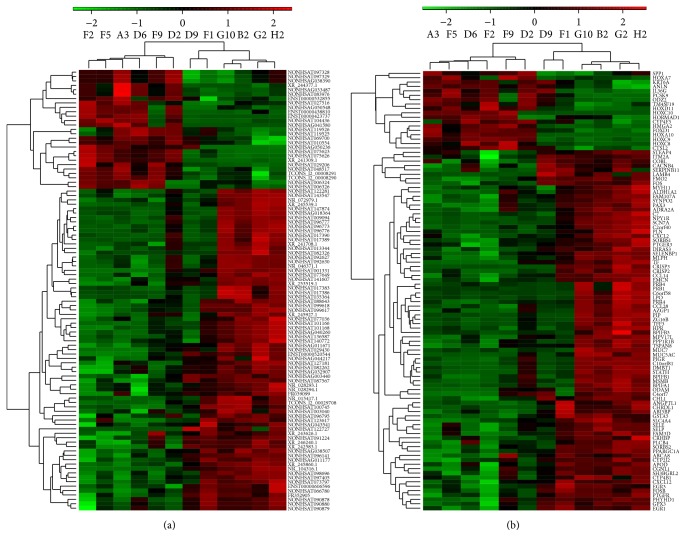
Heat map of lncRNAs and mRNAs that were often aberrantly expressed in SSCC compared with paired adjacent noncancerous sinonasal tissues. (a) Hierarchical clustering analysis of the top 100 dysregulated lncRNAs. (b) Hierarchical clustering analysis of the top 100 dysregulated mRNAs. Each row represents one lncRNA or mRNA, and each column represents one tissue sample. The relative lncRNA or mRNA expression is depicted according to the color scale. Red indicates elevated expression and green indicates reduced expression. 2, 0 and −2 are fold changes in the corresponding spectrum. Carcinoma group (A3, F5, D6, F2, F9, and D2). Paired adjacent noncancerous group (D9, F1, G10, B2, G2, and H2).

Hierarchical clustering of the lncRNAs and mRNAs profile was performed using cluster 3.0.2; hierarchical clustering of the expression of the top 100 dysregulated lncRNAs and top dysregulated 100 mRNAs based on centered Pearson correlation clearly separated SSCC tissues from corresponding normal tissues (Figure 2).

### 3.2. lncRNA-mRNA Coexpression Network

**Figure 3:**
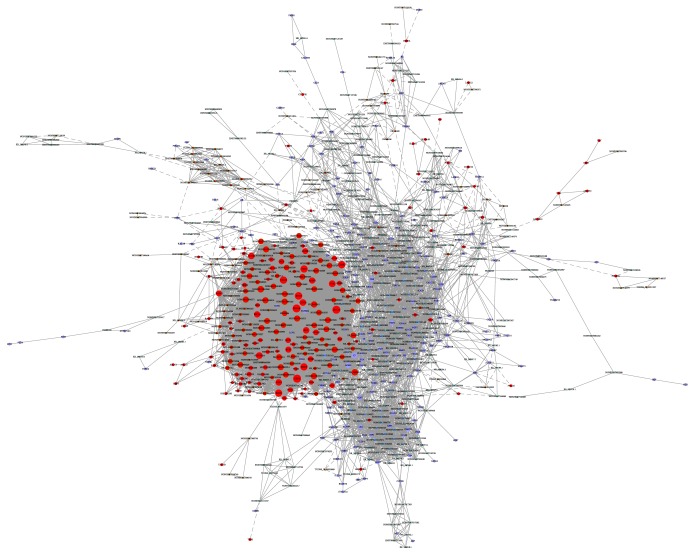
lncRNA-mRNA coexpression network. The SSCC consisted of coexpression relationships between lncRNAs and mRNAs. The red circles denote mRNAs and the blue circles denote lncRNAs. The node degree is indicated by the circle size. An edge represents a coexpression relationship between mRNA and a lncRNA in the context of SSCC progression.

We constructed the lncRNA-mRNA coexpression network to identify the interactions between mRNAs and lncRNAs. The results showed that the coexpression network was composed of 787 network nodes and 8478 connections between 445 lncRNAs and 342 mRNAs. Within this coexpression network, 7635 pairs connections presented as positive, and 843 pairs connections presented as negative (Figure 3). This coexpression network indicated that one lncRNA (NONHSAT041869) could target 118 mRNAs/mRNAs at most and one mRNA (EXO1) could correlate with 122 lncRNAs/mRNAs at most. The results implied that EXO1, CDCA5, and BUB1B may play key roles in SSCC process and development.

### 3.3. Function Analysis of Differentially Expressed Genes

**Figure 4:**
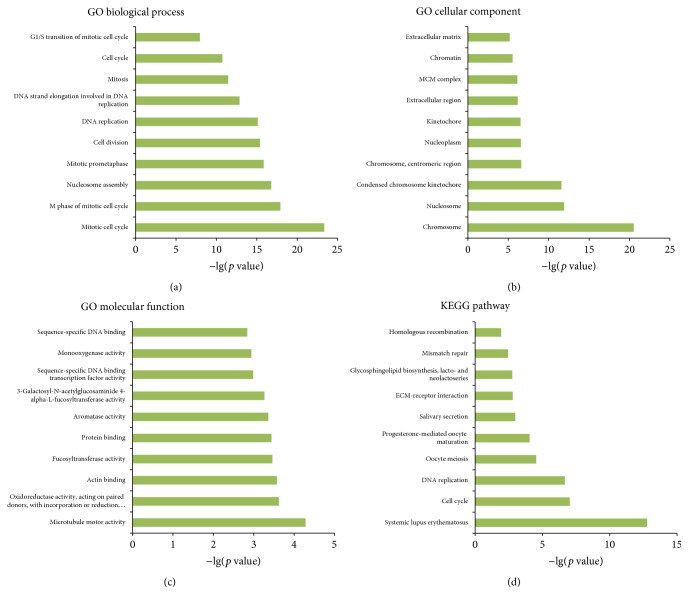
GO and KEGG pathway analysis. (a)–(c) Top 10 enrichment GO terms for differentially expressed mRNAs. The bar plot shows the enrichment scores (−lg(*p* value)) of the significant enrichment GO terms. (a) GO terms of biological process (BP); (b) GO terms of cellular component (CC); (c) GO terms of molecular function (MF). (d) Top 10 pathways of differentially expressed mRNAs in SSCC. The vertical axis represents the pathway category and the horizontal axis represents the enrichment score (−lg(*p* value)) of the pathway.

Functional roles of lncRNAs can only be indirectly predicted by analyzing the functions of their coexpressed mRNAs, because most lncRNAs' functions have not yet been defined. To investigate underlying biological associations, we ran GO and KEGG pathway analysis on the top 500 differentially expressed lncRNAs and mRNAs. GO analysis indicated that these differentially expressed genes were enriched in 12 biological processes; the majority were proven to be related to cancer-associated biological behaviors; the top 3 were multicellular organismal development, mitotic cell cycle, and cell cycle. The differentially expressed genes also were enriched in 12 cellular components; the top 3 were nucleus, extracellular region, and cytosol. Similarly, 12 molecular functions were enriched for including protein binding, DNA binding, and ATP binding. KEGG analysis revealed pathways associated with cancer, such as microRNAs in cancer, p53 signaling pathway, and PI3K-Akt signaling pathway (Figures 4(a)–4(d)).

### 3.4. Gene Signal-Network

**Figure 5:**
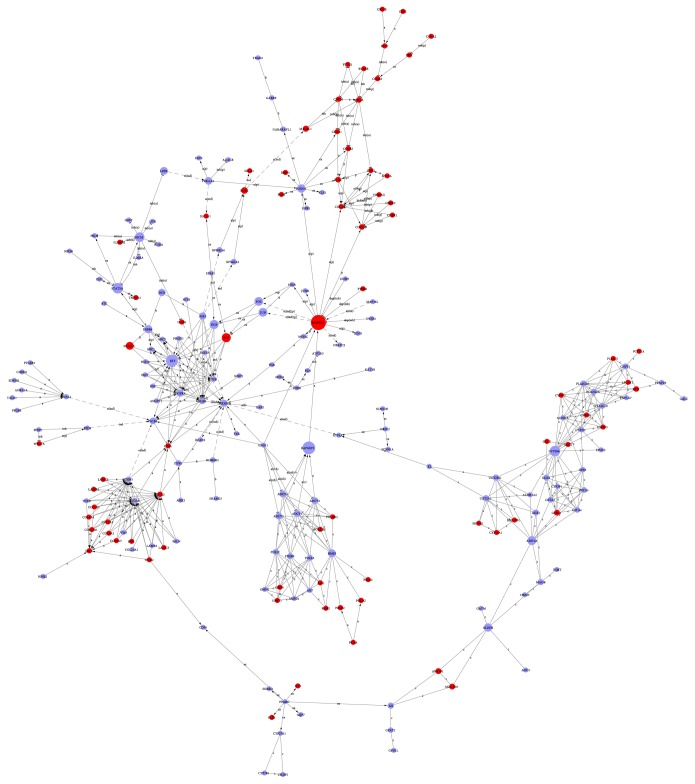
Signal-net. The interaction network of differentially expressed genes (signal-net). The circles represent important functional genes in SSCC (blue: downregulated genes; red: upregulated genes); the circle size represents the degree of interaction (betweenness centrality); the lines indicate the interactions.

We performed a signal-net analysis to investigate the global network, based on the significantly regulated KEGG. With signal-net, we screened the important dysregulated genes involved in the differences between SSCC and normal tissues (Figure 5). The results showed that the core genes may have played an important role in SSCC process. According to the results of this analysis, the top 3 betweenness genes were* MAPK12, RAPGEF3*, and* KIT.*

### 3.5. qRT-PCR Validation

**Figure 6:**
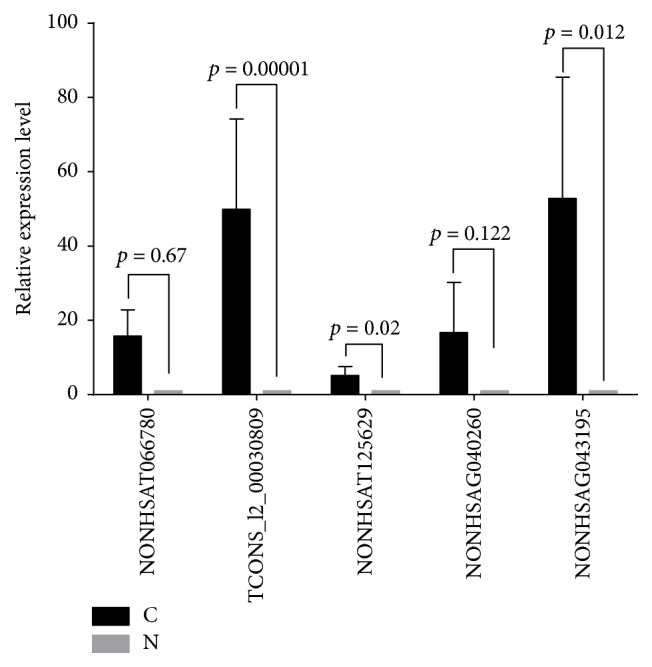
qRT-PCR validation. qRT-PCR verification of 5 candidate lncRNAs in 22 pairs of SSCC tissues. The *y*-axis represents the relative expression levels of lncRNAs. Paired *t*-tests (2-tailed) were performed to compare the expression levels between carcinoma (C) and noncancerous tissues (N), and a *p* value <0.05 indicated statistical significance.

Five differentially expressed lncRNAs were randomly selected for validation by means of qRT-PCR according to the manufacturer's recommendations. NONHSAT125629 and TCONS_l2_00030809 were upregulated and NONHSAT066780, NONHSAG040260, and NONHSAG043195 were downregulated in SSCC. The results of qRT-PCR were consistent with those of the microarray. All of the 5 lncRNAs were differentially expressed with the same trend (up- or downregulated) (Figure 6).

## 4. Discussion

SSCC is a rare disease arising in the epithelium of respiratory tract and is very poorly studied from the molecular perspective. To date, the pathogenesis of SSCC remains unclear due to its low incidence. Only a few studies have focused on its pathogenesis and potential molecular targets for therapy, specifically microRNAs [10–12]. Recently, studies have increasingly shown that many types of tumors are closely associated with the abnormal expression of lncRNAs [6–9]. In head and neck cancer, tongue cancer [13], laryngeal cancer [14], nasopharyngeal cancer [15], and thyroid cancer [6] are all associated with the abnormal expression of lncRNAs. However, to the best of our knowledge, there are no reports on lncRNA expression profiles in SSCC.

Here, we investigated the lncRNA and mRNA expression profiles of SSCC samples from patients using microarray analysis. We identified thousands of lncRNAs that are expressed significantly differently in SSCC compared to adjacent noncancerous tissues, including both upregulation and downregulation. To some extent, false positive results do exist in the microarray detection. Therefore, 5 lncRNAs were randomly selected to validate the microarray results. Consistent with the microarray results, all of the 5 lncRNAs were differentially expressed based on the results of qRT-PCR.

In this study, we used GO and KEGG pathway analyses to identify biological functions enriched among the differentially expressed mRNAs. We found that these mRNAs were involved in a lot of cancer-associated biological processes, cellular components, and molecular functions. The GO annotation indicated that these gene products may affect the tumorigenesis and development of SSCC. The KEGG pathway analysis identified that many pathways were related to cancer, such as microRNAs in cancer, P53 signaling pathway, PI3K-Akt signaling pathway. For example, it has been documented that the p53 signaling pathway is activated in many solid tumors, including SSCC [1, 16, 17]. In the present study, p53 signaling pathway was related to lncRNA NONHSAT125629. This molecule may participate in numerous biological processes, including mitotic cell cycle, cell division, DNA replication, G1/S transition of mitotic cell cycle, and G2/M transition of mitotic cell cycle.

The global network (gene signal-network) and lncRNA-mRNA network structure analysis were established to show the core genes that play a critical role in this SSCC gene network. However, how these genes participate in the pathogenesis of SSCC largely remains unknown. The analysis revealed that MAPK12, RAPGEF3, and KIT exhibited the most betweenness centrality and all were related to the cancer progression. Thus, our preliminary data provide a justification for the involvement of these genes in SSCC development. For example, KIT is associated with various pathways related to cancer, such as pathways in cancer and PI3K-Akt signaling pathway. Mutations in this gene are associated with gastrointestinal stromal tumors [18], lung cancer [19], and breast cancer [20].

The limitation of this study lies in the fact that the sample size is relatively small. Our results require further validation in larger prospective patient cohorts and functional experiments, both* in vitro* and* in vivo*. Our results provide some valuable clues for future function and mechanism studies of SSCC.

## 5. Conclusions

In summary, to our knowledge, our study is the first screening and analysis of lncRNA expression profile in SSCC. The results show that genes regulated by these lncRNAs are involved in cancer pathways as a proof of principle. This may offer new insights into pathogenesis and could be a promising way to dissect the molecular pathogenesis of this refractory cancer. Our study lays the foundation for further investigation of this disease. Further large scale studies are warranted to provide convincing evidence for clarifying the functions of lncRNAs in SSCC and determining whether these lncRNAs can serve as new diagnostic biomarkers, prognostic factors for survival, and therapeutic targets in SSCC.

## Figures and Tables

**Table 1:** Clinical parameters of 6 SSCC patients that underwent lncRNA expression profiling.

Specimen number of SSCC tissues	Gender	Age (years)	Exposure factor	TNM stage	Histologic differentiation	Specimen number of noncancerous tissues
F5	Male	81	Wood dust	T1N0M0	Well	G2
F9	Male	46	Tobacco smoke	T3N0M0	Poorly	G10
A3	Male	52	Tobacco smoke	T4N0M0	Moderately	B2
D6	Male	72	Wood dust	T4N0M0	Moderately	F1
F2	Male	52	Tobacco smoke	T3N0M0	Moderately	H2
D2	Male	66	Tobacco smoke	T3N0M0	Moderately to Poorly	D9

**Table 2:** The primer sequences in the present study.

lncRNA name	Forward primers (5′-3′)	Reverse primers (5′-3′)	Amplicon size (bp)
NONHSAT066780	GGAACCAGCTACTCCACACC	CCTACCCAGGCCAAGTTCTG	182
TCONS_l2_00030809	TGAAAAACCACAGGCCCACT	ACAAACATGTCTCTCATCAGCAC	78
NONHSAT125629	GATTTGAATCGGTCGGCGG	AGGCATTTCCTCTCACGCC	284
NONHSAG040260	ATGCCCTACGAATGTGGACC	TCGGCCCACTGCTAAACATC	218
NONHSAG043195	GGGAAGGCTGCCTATGAAGG	AATTCGGGGTTGCAGGTTCT	184

**Table 3:** Top 20 aberrantly expressed lncRNAs in microarray for 6 pairs of SSCC and paired adjacent noncancerous sinonasal tissues.

Probe name	*p*	FC (abs)	Regulation	ncRNA accession	Gene symbol	Chr
CUST_13287_PI429545395	0.019137	239.3843	Down	NONHSAT096777	STATH	chr4
CUST_21727_PI429545388	0.039463	84.90894	Down	NONHSAT009094	PIGR	chr1
CUST_76033_PI429545399	0.024086	82.32558	Down	NONHSAT013344	MSMB	chr10
CUST_5401_PI429545406	0.024154	78.48107	Down	NONHSAG018364	NONHSAG018364	chr16
CUST_82017_PI429545376	9.06*E* − 04	76.64131	Down	TCONS_l2_00029708	linc-RUSC2	chr9
CUST_13257_PI429545395	0.019957	75.85599	Down	NONHSAT096773	STATH	chr4
CUST_13277_PI429545395	0.023234	74.90795	Down	NONHSAT096776	STATH	chr4
CUST_30951_PI429545395	9.49*E* − 05	67.20975	Down	NONHSAT100745	CTD-2351A8.1	chr5
CUST_91115_PI429545399	0.04924	65.20921	Down	NONHSAT017390	MUC5B	chr11
CUST_5411_PI429545406	0.020191	56.05385	Down	NONHSAT147874	ZG16B	chr16
CUST_17568_PI429545406	0.02086	53.66862	Down	NONHSAT143547	HP	chr16
CUST_32391_PI429545395	0.001238	51.26765	Down	NONHSAT101166	C7	chr5
CUST_57295_PI429545380	1.32*E* − 04	49.87698	Down	FR352905	FR352905	chr19
CUST_15837_PI429545395	7.74*E* − 04	47.76275	Down	NONHSAT097405	MMRN1	chr4
CUST_32401_PI429545395	0.001893	41.53056	Down	NONHSAT101168	C7	chr5
CUST_58955_PI429545388	1.03*E* − 05	40.74976	Up	NONHSAT075623	HOXD11	chr2
CUST_72258_PI429545388	2.00*E* − 04	39.85786	Down	NONHSAT087567	CHL1	chr3
CUST_58985_PI429545388	5.09*E* − 06	38.35	Up	NONHSAT075626	HOXD10	chr2
CUST_32370_PI429545395	0.007453	33.21905	Down	NONHSAG040260	NONHSAG040260	chr5
CUST_24853_PI429545410	0.003046	31.34336	Down	XR_253519.1	LOC101928556	N/A

FC, fold change; Chr, chromosome; N/A, not annotated.

**Table 4:** Top 20 aberrantly expressed mRNAs in microarray for 6 pairs of SSCC and paired adjacent noncancerous sinonasal tissues.

Probe name	*p*	FC (abs)	Regulation	Gene symbol	Chr	Genbank accession
A_33_P3265783	0.015708	379.6405	Down	STATH	chr4	NM_003154
A_23_P154784	0.043979	159.44	Down	BPIFB1	chr20	NM_033197
A_33_P3300312	0.034496	146.1192	Down	DMBT1	chr10	NM_007329
A_24_P844984	0.030927	119.5701	Down	PIGR	chr1	NM_002644
A_23_P86599	0.023373	95.27439	Down	DMBT1	chr10	NM_007329
A_24_P146683	0.032317	89.55136	Down	MSMB	chr10	NM_002443
A_33_P3216570	0.003716	75.59971	Down	MUC5AC	N/A	AJ298317
A_23_P218369	3.92*E* − 04	73.96607	Down	CCL14	chr17	NM_032963
A_23_P118203	0.017284	68.04929	Down	ZG16B	chr16	NM_145252
A_21_P0013344	0.032196	61.89528	Down	AZGP1	chr7	NM_001185
A_23_P95930	6.01*E* − 05	58.4917	Up	HMGA2	chr12	NM_003483
A_32_P173662	0.002064	58.24841	Down	CRISP2	chr6	NM_003296
A_23_P362694	0.036618	54.65685	Down	C4orf7	chr4	NM_152997
A_33_P3245228	0.031988	52.29204	Down	BPIFA1	chr20	NM_130852
A_23_P58228	0.017304	50.18366	Down	ODAM	chr4	NM_017855
A_23_P429998	1.13*E* − 04	48.27517	Down	FOSB	chr19	NM_006732
A_33_P3233040	0.012252	42.21305	Down	SERPINB11	chr18	NM_080475
A_23_P8702	0.018377	40.33374	Down	PIP	chr7	NM_002652
A_33_P3275702	1.49*E* − 05	33.86336	Down	FMO2	chr1	NM_001460
A_21_P0000003	0.006532	31.00913	Down	PRR4	chr12	NM_007244

FC, fold change; Chr, chromosome; NA, not annotated.

## Abbreviations

lncRNA: Long noncoding RNA
SSCC: Sinonasal squamous cell carcinoma
qRT-PCR: Quantitative real-time polymerase chain reaction
GO: Gene ontology
KEGG: Kyoto Encyclopedia of Genes and Genomes
TNM: Tumor-node-metastasis
AJCC: American Joint Committee on Cancer
FC: Fold change
Chr: Chromosome
N/A: Not annotated.
